# Maternal effects on reproduction in the precocial European hare (*Lepus europaeus*)

**DOI:** 10.1371/journal.pone.0247174

**Published:** 2021-02-17

**Authors:** Stéphanie C. Schai-Braun, Peter Steiger, Thomas Ruf, Walter Arnold, Klaus Hackländer

**Affiliations:** 1 Institute of Wildlife Biology and Game Management, University of Natural Resources and Life Sciences, Vienna, Austria; 2 Research Institute of Wildlife Ecology, University of Veterinary Medicine, Vienna, Austria; 3 Deutsche Wildtier Stiftung—German Wildlife Foundation, Hamburg, Germany; Institute of Animal Science, CZECH REPUBLIC

## Abstract

In female mammals, reproduction, and in particular lactation, is the energetically most exigent life-history phase. Reproduction is strongly controlled by body reserves and food availability, so females with better body condition or food supply are believed to have higher reproductive output. Additionally, the growth and mortality of young mammals depends on their postnatal development. Therefore, the degree of precociality affects energetic demands for both mothers and young. To study the reproductive performance of the precocial European hare (*Lepus europaeus*), we analysed relationships between six predictor variables describing maternal and environmental effects and nine response variables relating to reproduction from 217 captive females. We compared the data with those of precocial and altricial mammal species from an extensive literature search. For hares, we found: (1) Heavier females had heavier litters at birth. (2) In summer and spring, total litter mass was larger than in winter. (3) At the end of lactation, the litters of multiparous females were heavier than those of primiparous females. (4) Both older females and females giving birth for the first time had relatively high leveret mortality during lactation. Comparing our results with the literature for other mammals revealed that the body condition (i.e., body mass) of females before birth is predictive of reproductive parameters in both precocial and altricial species. In the precocial hare, female body condition is no longer predictive of reproductive parameters at the end of lactation, whereas in altricial species, female body condition remains predictive of reproduction (litter mass at the end of lactation, offspring mortality) until the end of lactation. We conclude that these effects are caused by precocial offspring feeding on solid food soon after birth and, thus, being less dependent on the mother’s body condition during lactation than altricial offspring. In line with this, precociality might have evolved as a way of buffering offspring against maternal effects.

## Introduction

Reproduction is a costly process [1–4] and is directly linked to the evolution of reproductive strategies [5] and population dynamics [6]. In female mammals, reproduction is the most energetically demanding life-history phase [7], so it is firmly regulated by body condition and food availability [8]. Of the reproductive processes, lactation is the most energetically demanding for female mammals; it results in maximum metabolic rates and energetic costs [1, 2, 9, 10]. Females in optimal body condition are able to make high reproductive investments without impairing their future survival and reproduction [11, 12]. In several mammalian species, offspring mass is indeed positively correlated with the mother’s body mass [13–16] although fathers have an influence on offspring mass too due to their contribution to the offspring genotype. However, reproduction can reduce the mother’s body condition [17–20] and, hence, future reproductive performance [21–23]. Two strategies in maternal investment have evolved: capital breeders use their own body fat reserves to cover the high energetic costs of reproduction, whereas income breeders increase their energy or food intake [24]. Questions relating energy to reproductive performance are difficult to address in wild mammals. In captive females, it is possible to vary diet composition in order to quantify the influence of energy availability, female body mass and length of lactation on reproductive output (i.e. the number of young born) and reproductive success (i.e. the number of young that survive until the end of lactation).

Reproductive performance in wild female mammals may vary seasonally. Due to seasonal fluctuations in climate and day length, ecosystems at higher latitudes and in continental habitats are characterized by large seasonal changes in ecologically important parameters such as temperature and the availability of food [25]. Correlated with these variations, most mammals dwelling in such habitats show seasonal changes in breeding activity [25, 26], including seasonal variations in litter size [27–29] and offspring body mass at birth [30, 31].

Further, fertility rate is typically low at the beginning of the reproductive life of a female mammal [32], increases thereafter and starts to decrease again from about the middle of the reproductive span onwards [32, 33]. However, age-related variation in fertility is almost impossible to study in wild populations because the number of senescent individuals is low, due to predation and disease [34–36].

The growth and mortality of young mammals depend not only on characteristics of their mother, but also on their postnatal development. Two strategies have evolved in mammals–altricial and precocial postnatal development–such that a continuum exists between them. Precocial young are born, after a prolonged gestation period, with fur and open eyes, and are well-developed and mobile soon after birth. In contrast, altricial young are born without fur and with closed eyes, are rather immobile, and have a long postnatal period of parental dependency. In mammals, altricial young are more common, but precocial young occur in numerous mammalian taxa [37]. Small mammals generally have large litters of altricial young and high reproductive rates, whereas large mammals usually produce precocial young in small litters [37]. Female precocial rodents have a lower peak energy demand during lactation than female altricial rodents, as precocial offspring start to take solid food earlier [38].

In both precocial and altricial reproduction, output (number of offspring) is positively correlated with female body mass (e.g., [9, 39, 40]), decreases in older females (e.g., [33]) and varies during the year in seasonal environments (e.g., [41]). Both female characteristics (e.g., body mass at mating, age, parturition sequence, lactation duration) and environmental characteristics (e.g., season of birth, food availability) can affect the growth and mortality of young in both precocial and altricial species during the lactation period. However, little is known about the influence of female and environmental characteristics on reproductive parameters (e.g., litter size, litter mass, mean body mass of offspring) at the end of lactation in precocial and altricial mammals, and the trade-offs associated with the different strategies of postnatal development during the lactation period are not well understood. Especially in precocial mammals, reproductive parameters at the end of lactation are scarce (but see [30, 42]).

This study focuses on the European hare (*Lepus europaeus*), a precocial polygynandrous species without social hierarchy [43, 44]. Several previous studies described reproductive biology and strategy in this lagomorph, but important questions remain unanswered. Hackländer et al. [45] showed that female diet during lactation affects the energy budget of mothers, but does not influence total offspring body mass gain during lactation. Litter size within each reproductive season increases from the first litter and then declines towards the last litter in wild European hares [46–50], but whether season of birth influences leveret body mass and total litter mass is unknown. Offspring body mass at birth is decisive for offspring survival [51–54], especially in precocial mammalian species that reach maternal independence rather early [51]. Moreover, the fertility of female European hares depends on age. Most leverets are born during a mother’s third year of life. After this peak, fertility decreases and senescence takes place even if body condition is high [48, 55]. It is important to understand the influence of maternal age on reproductive parameters such as litter size, leveret body mass, and litter mass, in order to quantify maternal effects in the precocial hare.

The goal of this study was to investigate the reproductive performance of female European hares and to compare the results with data from other precocial and altricial mammals. Our hypotheses were: (1) female hares’ diet and body mass affect females’ reproductive output and success; (2) litter mass is highest and leveret body mass is lowest in the middle of the breeding season; (3) reproductive parameters that are decisive for the reproductive performance (i.e. litter size, litter mass, leveret body mass and leveret survival during lactation) are lower in primiparous females and in females at the end of their reproductive life; and (4) in European hares and other precocial mammals, but not in altricial mammals, female and environmental characteristics affect reproductive parameters at birth and at the end of lactation in different ways (e.g. female body mass is only relevant until birth, whereas the season is relevant until the end of lactation). To test these hypotheses, we analysed data from captive female European hares and conducted an extensive literature search on reproductive parameters of precocial and altricial mammals.

## Material and methods

### Captive hares and data collection

Our study animals were born, individually marked and maintained in the outbred breeding colony of European hares at the Research Institute of Wildlife Ecology in Vienna (Austria). The study was discussed and approved by the institutional ethics and animal welfare committee of the University of Veterinary Medicine, Vienna, in accordance with GSP guidelines and national legislation. Data were collected over a period of 16 years (1989–2004). The hares were kept in outdoor cages with wire-netting floors (0.5 m^2^). They were sheltered from the wind and direct sunlight, but exposed to the natural photoperiod and ambient temperatures (mean annual air temperature: 10.4°C, annual minimum air temperature: -19.6°C, annual maximum air temperature: 37.6°C, data provided by the Austrian Central Institute for Meteorology and Geodynamics, weather station Wien Hohe Warte, 48°14’N, 16°21’E). All individuals had access to food and water *ad libitum*, and were given regular anti-parasitic treatment. Generally, males, females, and leverets were kept separately. Only for mating, a male was placed into a female’s cage for two nights. Males were selected randomly in order to avoid bias due to the influence of male body condition on leveret body condition. Females that did not mate successfully were removed from the breeding program; this mostly happened when they were between four and six years old. Animals were housed for use in further research following this research. Because leverets are nursed only once a day in the wild [56], we separated them from their mothers except for between 08:00 and 09:00 h each day, when they were placed into their mother’s cage and were allowed to suckle. Females always accepted their young for suckling irrespective of any potential disturbing factor, e.g. human odour. We stopped putting leverets with their mothers for suckling after four to five weeks, depending on each female’s level of aggression towards her offspring and the body mass of the leverets. This period of weaning corresponds to that found in the wild [56].

At birth, and at the end of lactation, litter mass was measured by weighing the entire litter, and mean leveret body mass was determined by dividing litter mass by litter size. All weights were taken to the nearest gram. Leverets were sexed according to secondary sexual characteristics. Sex ratio at birth was calculated by the number of male leverets at birth divided by the litter size at birth. Leveret mortality during lactation was determined by litter size at the end of lactation subtracted from litter size at birth divided by litter size at birth. The age of each hare was known as all birth dates were recorded.

We determined female mass and age at mating. Lactation duration was defined as the number of days leverets were put with their mothers for suckling. Female hares were in two diet groups: one was fed with standard hare pellets (‘low-fat diet’, Raiffeisen, Salzburg, n = 157; number of litters = 947) and the other was fed with high-fat pellets (‘high-fat diet’, n = 77; number of litters = 473). Note that 17 females changed diet group once but never during reproductive season. The nutrient composition of the low-fat diet corresponded to chemical analysis of stomach contents from free-ranging hares [57, 58]. The high-fat diet was produced by adding 1 kg of sunflower oil that was rich in fatty acids to 10 kg standard pellets. This procedure resulted in a profound increase in dietary fat content (for further information see [45]). We defined ‘season of birth’ as winter (December to February), spring (March to May), summer (June to August), or autumn (September to November).

### Data analysis

Data were collected partly repeatedly from the same females in different years. Therefore, we used generalized linear models (GLMMs), with separate intercepts for females, nested into each year, as random effects. This allowed us to adjust for repeated measurements from a female and under different environmental conditions in each study year. GLMMs were computed using R [59] packages ‘brms’ [60] and ‘rstan‘ [61]. The Bayesian GLMM approach implemented in these libraries has the advantage that it can readily estimate random effects even when data are obtained partly as repeated measures, and partly from a single reproductive bout in one female. This data structure often causes singularities and prevents random effect estimates with other methods. Multicollinearity between the predictor variables was checked calculating the Variance Inflation Factor (VIF) with R package ‘car’ [62] for all independent variables in each model. The variables litter size at birth and litter mass at birth had a VIF>8. Therefore, we removed litter size at birth from the one full model including these two predictors.

All GLMM samples were drawn with the NUTS algorithm using four chains and at least 4000 iterations (6000 for more complex models). We only report models that converged with all Rhat<1.05.

In addition to the above random effects, all full models included the following five predictor variables describing maternal and environmental effects: female mass at mating, female age (both variables scaled), reproductive state (primiparous/multiparous), female diet (high/low fat) and season of birth (factor with levels winter, spring, summer, autumn) as well as the two-way interactions between these variables. Some models included additional fixed predictors (for an overview of all models see S1 Table). We eliminated model terms to determine the model that minimized leave-one-out cross-validation Information Criterion (LOOIC; [63]) using the R package ‘loo’ [64]. As a measure of support for the inclusion of a variable, we used the increment of the increase caused by its removal, Δ LOO.

The response variables litter size at birth and litter size at the end of lactation were Poisson distributed without overdispersion (inspected with library ’fitdistrplus’, [65]), and were analysed accordingly. For the response variables litter mass at birth and mean leveret body mass at birth, we used the family function ‘gaussian’. GLMMs for normally distributed data were also used to model the mass gain of litters and mean body mass gain of leverets during lactation. The response variables in these models were litter mass and mean leveret body mass at the end of lactation, which were always adjusted for litter mass or mean leveret body mass at birth, entered as fixed predictors. Litter mass and body mass gain were only analysed for litters in which there was no mortality to avoid the effect of a sudden change in sibling rivalry on the response variables.

Sex ratio and leveret mortality (during lactation) were analysed using binomial models. For each reproductive bout, we considered litter size as the ‘trial’ number, and the number of male leverets (for the sex ratio) or the number of leverets dying (for mortality) as the ‘incidence’ number. We used only flat (uniform) prior distributions, as we had no prior information on expected slopes, and because this avoids bias on the resulting posterior distributions [66, 67].

To test the effect of female age on milk quantity, we reanalysed data on 21 females published by Hackländer et al. [45] with Bayesian GLMM similar to the other analyses of this study. The daily milk quantity was examined using the family function ‘gaussian’. The full model included female age, litter size, leveret age (in weeks), season of birth, and female diet (low- or high-fat), with intercepts for females, nested into each year, as random effects.

### Literature search

The Web of Science (Clarivate Analytics, Boston, MA, USA) was used as a research tool for finding scientific articles on mammalian reproduction (date limit: 30.06.2020). Search terms were the parameters used to describe reproduction (i.e. litter size, mean young mass, litter mass, young mortality, lactation duration) in combination with mammals. Furthermore, terms describing postnatal development (e.g., altricial, precocial), maternal effects (i.e. female body mass, female age, parturition sequence, lactation duration) and effects of environmental characteristics (i.e. season of birth, diet availability) were included in the literature search. We included synonyms of the search terms (e.g., offspring, young).

## Results

We analysed data on reproductive performance of 217 females producing 909 leverets in 442 litters. Female mass at mating ranged from 2210 g to 4905 g with a mean of 3562 g (± 13.42 SE). The youngest female to give birth produced her litter at the age of 109 days, so she had mated successfully at the very young age of about two months (67 days). This female was born in April and had her first litter of only one leveret in July (leveret’s body mass at birth: 124 g). The oldest female to give birth produced her litter at the age of 2317 days (~ 6.3 years). On average, females gave birth for the first time at the age of 423 days (± 27.34 SE, ~ 1 year and 2 months old), and for the last time at the age of 740 days (± 43.85 SE; about two years old). The sex ratio of offspring was balanced (at birth: 51% males; at the end of lactation: 48% males).

### Reproductive performance

**Fig 1:**
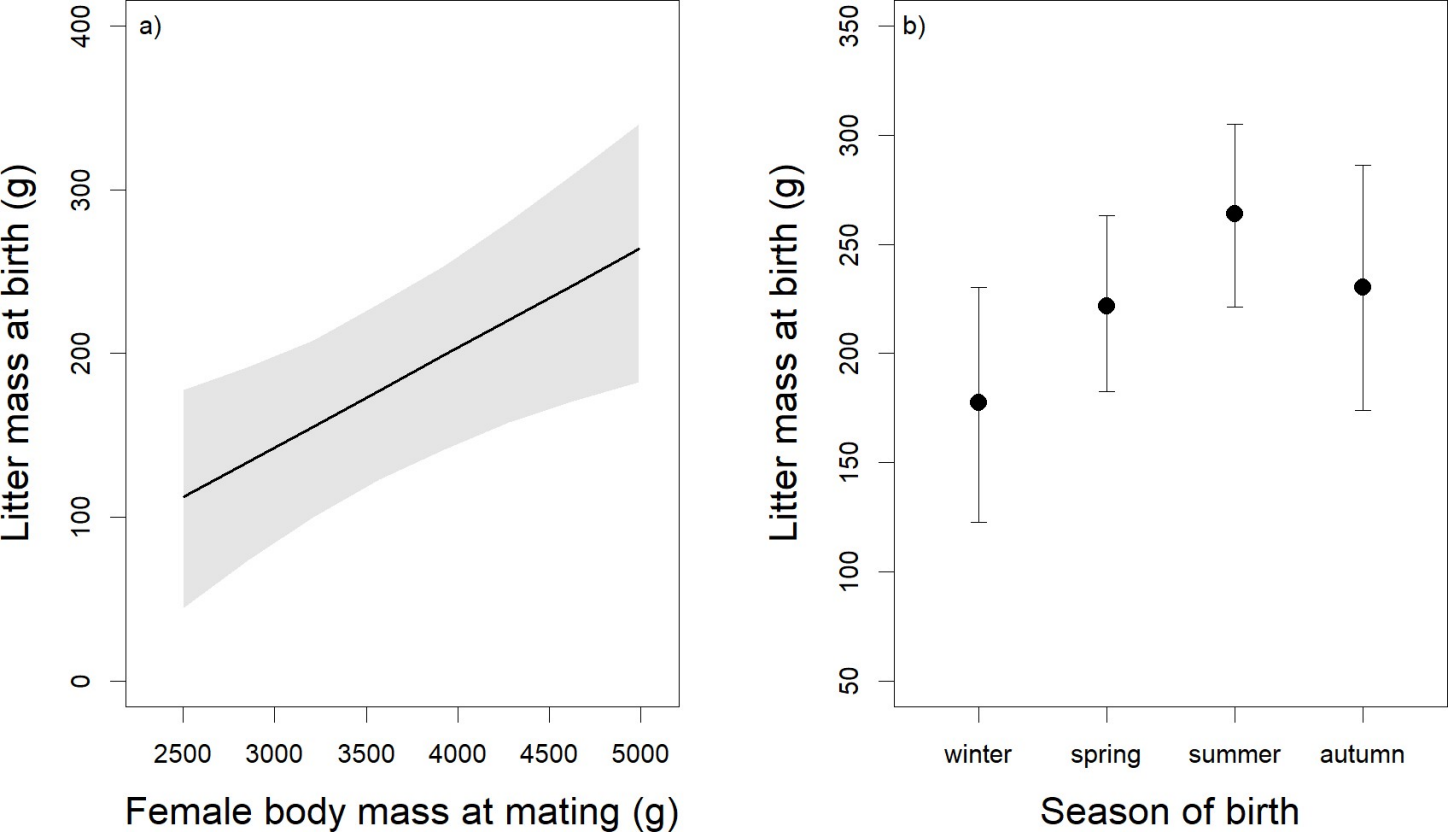
Relationships between a) female body mass at mating and litter mass at birth and b) season of birth and litter mass at birth. Relationships between a) female body mass at mating and litter mass at birth, with the line representing the predicted median values and the grey area displaying the Bayesian credible intervals; and b) season of birth and litter mass at birth, with the points representing the predicted median values and the whiskers displaying the Bayesian credible intervals (n = 52; number of litters = 174). See text for details on statistics.

The coefficients of the best model for each of the nine response variables are shown in Table 1. Litter size at birth was not closely associated with by any predictor variable. However, the best model suggested that heavier females had larger litters (-0.03 to 0.19; β = 0.08; Δ LOO 0.8; for), but litter size at birth showed no strong seasonal effect (litter size at birth in winter: mean = 1.65 ± 0.15 SE; spring: mean = 1.77 ± 0.09 SE, summer: mean = 2.13 ± 0.11 SE; autumn: mean = 1.94 ± 0.21 SE). Heavier females had heavier litters at birth (β = 21.51; Δ LOO 4.7; Fig 1A), and litter mass at birth was related to the season of birth (Δ LOO 10.0): it was lower in winter and higher in spring and summer (Fig 1B). Mean leveret body mass at birth was lower in larger litters (β = -8.71; Δ LOO 29.9; Table 2), and leverets of primiparous females were lighter at birth than leverets of multiparous females (β = -9.94; Δ LOO 7.2).

**Table 1:** Coefficients of the best models for all nine response variables describing reproduction.

**Litter size at birth**	**Estimate**	**Est. Error**	**95% Credible Interval**
Intercept	0.61	0.10	[0.40, 0.80]
Female body mass at mating	0.08	0.05	[-0.03, 0.19]
**Litter mass at birth**	**Estimate**	**Est. Error**	**95% Credible Interval**
Intercept	176.58	26.91	[123.72, 228.40]
Female body mass at mating	21.43	6.82	[8.04, 34.60]
Season of birth: Spring	45.65	23.19	[0.16, 90.68]
Season of birth: Summer	87.07	22.80	[42.17, 131.38]
Season of birth: Autumn	53.91	28.21	[-1.25, 108.96]
**Mean leveret body mass at birth**	**Estimate**	**Est. Error**	**95% Credible Interval**
Intercept	130.36	2.49	[125.54, 135.12]
Litter size at birth	-8.71	1.61	[-11.81, -5.54]
Reproductive state	-9.94	3.58	[-16.99, -3.06]
**Litter size at the end of lactation**	**Estimate**	**Est. Error**	**95% Credible Interval**
Intercept	0.50	0.10	[0.31, 0.69]
Litter size at birth	0.33	0.07	[0.19, 0.46]
**Litter mass at the end of lactation**	**Estimate**	**Est. Error**	**95% Credible Interval**
Intercept	0.11	0.07	[-0.03, 0.25]
Female diet	-0.14	0.09	[-0.31, 0.03]
Lactation duration	0.26	0.05	[0.16, 0.35]
Litter mass at birth	0.64	0.11	[0.42, 0.85]
Litter size at birth	0.31	0.11	[0.10, 0.53]
Reproductive state	-0.26	0.09	[-0.44, -0.086]
**Mean leveret body mass at the end of lactation**	**Estimate**	**Est. Error**	**95% Credible Interval**
Intercept	0.09	0.08	[-0.06, 0.23]
Lactation duration	0.24	0.05	[0.14, 0.34]
Mean leveret body mass at birth	0.92	0.04	[0.84, 1.00]
Reproductive state	-0.21	0.10	[-0.40, -0.02]
**Sex ratio at birth**	**Estimate**	**Est. Error**	**95% Credible Interval**
Intercept	-0.06	0.20	[-0.44, 0.35]
Female body mass at mating	-0.02	0.13	[-0.29, 0.23]
**Leveret mortality during lactation**	**Estimate**	**Est. Error**	**95% Credible Interval**
Intercept	-1.60	0.53	[-2.74, -0.66]
Female age	0.54	0.30	[-0.03, 1.18]
Reproductive state	1.41	0.50	[0.45, 2.41]
**Daily milk quantity**	**Estimate**	**Est. Error**	**95% Credible Interval**
Intercept	44.46	23.64	[-7.01, 89.06]
Leveret age	7.68	0.62	[6.49, 8.90]
Litter size at birth	13.69	1.52	[10.63, 16.61]
Season of birth: Spring	-7.41	3.69	[-14.63, -0.23]
Season of birth: Summer	1.31	3.34	[-5.36, 7.92]
Season of birth: Autumn	13.90	12.27	[-9.87, 37.71]

Coefficients of the best models for the response variables litter size at birth (n = 54; number of litters = 178), litter mass at birth (n = 52; number of litters = 174), mean leveret body mass at birth (n = 52; number of litters = 174), litter size at the end of lactation (n = 40; number of litters = 99), litter mass at the end of lactation (n = 40; number of litters = 98), mean leveret body mass at the end of lactation (n = 40; number of litters = 98), sex ratio at birth (n = 50; number of litters = 162), leveret mortality during lactation (n = 54; number of litters = 178), and daily milk quantity (n = 21, litters = 23).

**Table 2:** Mean leveret body mass at birth of leverets from different litter sizes.

Litter size at birth	Mean leveret body mass at birth (g)	SE	n
1	125.5	2.54	118
2	118.6	1.73	152
3	108.8	2.00	96
4	102.8	4.34	18
5	89.5	6.50	2

Mean leveret body mass, standard error and sample size of different litter sizes at birth (number of females = 114; number of litters = 386). Mean leveret body mass was determined by dividing litter mass by litter size.

**Fig 2:**
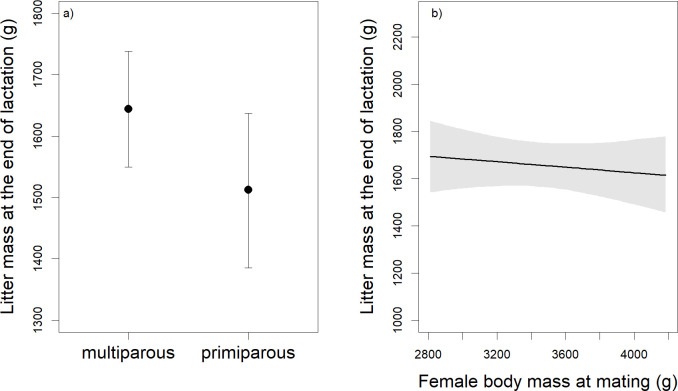
Relationships between a) female reproductive state and litter mass at the end of lactation and b) female body mass at mating and litter mass at the end of lactation. Relationships between a) female reproductive state and litter mass at the end of lactation, with the points representing the predicted median values and the whiskers displaying the Bayesian credible intervals (n = 40; number of litters = 98); and b) female body mass at mating and litter mass at the end of lactation, with the line representing the predicted median values and the grey area displaying the Bayesian credible intervals. See text for details on statistics.

Litter mass at the end of lactation was positively associated with lactation duration (β = 0.24; Δ LOO 17.5), and with litter mass at birth (β = 0.92; Δ LOO 184.9). Litters of primiparous females were lighter at the end of lactation than those of multiparous females (β = -0.21; Δ LOO 4.4; Fig 2A), but, by the end of lactation, litter mass was no longer related to female body mass at mating as it had been at birth (Fig 2B) and the effect of the season of birth was no longer significant. By the end of lactation, leverets of primiparous females remained lighter than those of multiparous females (β = -0.36; Δ LOO 2.4). Mean leveret body mass at the end of lactation was also positively associated with lactation duration (β = 0.52; Δ LOO 22.9) and with mean leveret body mass at birth (β = 0.42; Δ LOO 22.0).

**Fig 3:**
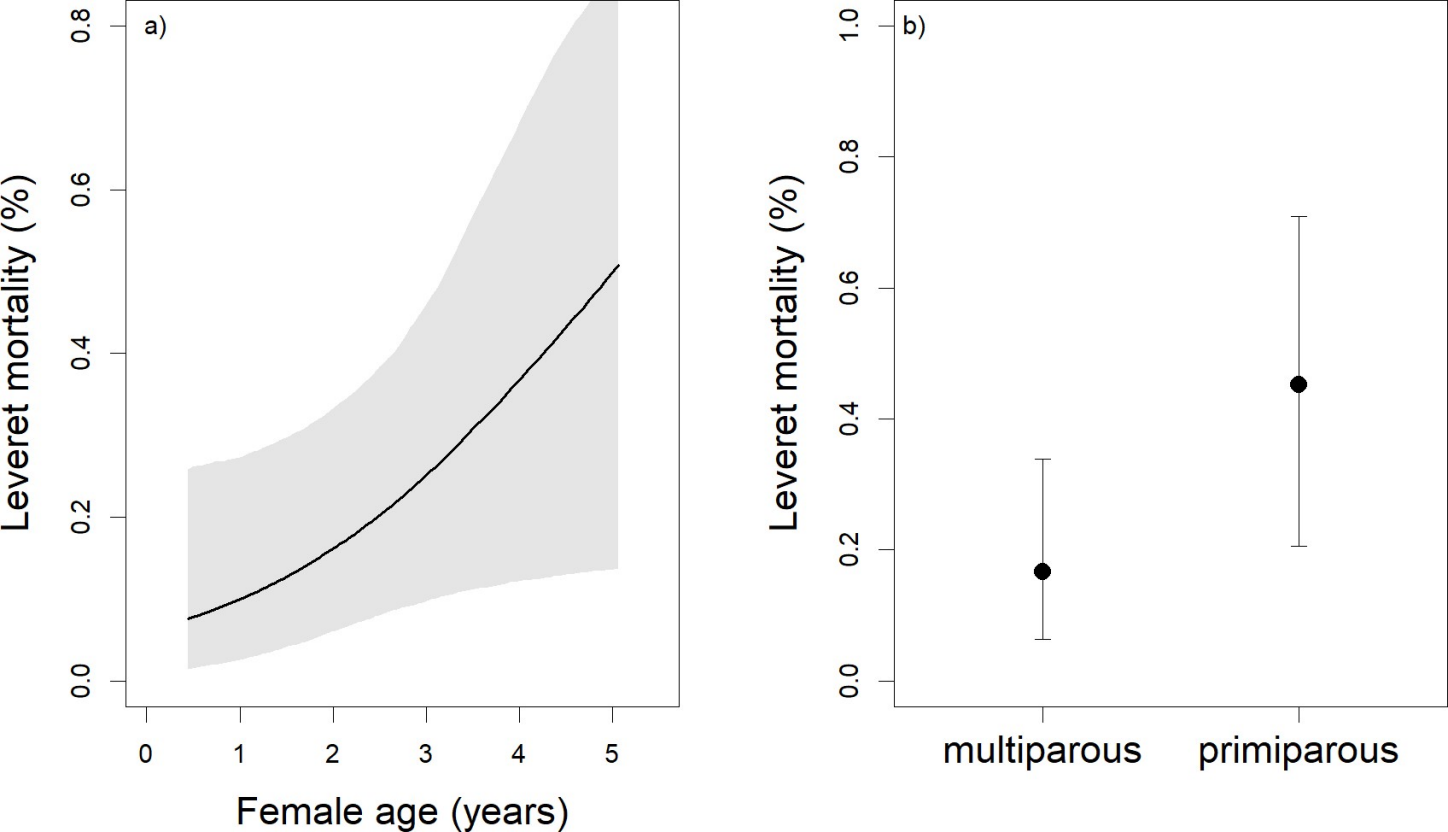
Relationships between a) female age and leveret mortality and b) female reproductive state and leveret mortality. Relationships between a) female age and leveret mortality, with the line representing the predicted median values and the grey area displaying the Bayesian credible intervals; and b) female reproductive state and leveret mortality, with the points representing the predicted median values and the whiskers displaying the Bayesian credible intervals (n = 54; number of litters = 178). See text for details on statistics.

Sex ratio at birth was not related to female body mass at mating, season of birth, female age, female diet or female reproductive state (all credible intervals including 0). Mean leveret mortality between birth and the end of lactation was 0.34. Leverets of older females (β = 0.55; Δ LOO 3.2; Fig 3A) and primiparous females (β = 1.42; Δ LOO 9.3; Fig 3B) had higher mortality during lactation than those of other females.

### Milk production

The daily milk quantity provided to offspring during lactation increased with litter size (β = 13.69; Δ LOO 48.4) and leveret age (β = 7.68; Δ LOO 142.1), and was lower for litters born in spring than for those born in winter (Δ LOO 7.0). Daily milk quantity was not significantly related to female diet (low- or high-fat) or female age.

### Literature search

Out of 71 articles on mammalian reproduction, we found 36 published articles in which authors provided information on reproductive parameters (i.e. litter size at birth, mean offspring mass at birth, litter mass at birth, litter size at the end of lactation, mean offspring mass at the end of lactation, litter mass at the end of lactation, offspring mortality between birth and the end of lactation; S2 Table) of mammals within the altricial and precocial postnatal development continuum (excluding parent-clinging mammals such as primates). Among them, 31 articles also included information on maternal effects (i.e., female body mass, female age, parturition sequence, lactation duration) and/or effects of environmental characteristics (i.e., season of birth, diet availability) on reproductive parameters (S3 Table). Among them, 22 articles analysed relationships between female body mass and reproduction (Table 3). No other maternal or environmental parameter was analysed regarding its effect on reproduction in the 36 published articles. Note that none of the studies on species near the middle of the continuum provided information on relationships between female body mass and reproduction. Hence, Table 3 only includes studies on precocial and altricial mammal species.

**Table 3:** Comparison of studies investigating relationships between female body mass and reproductive parameters in precocial and altricial mammal species.

	Influence	Precocial		Altricial	
		Study	Species	Study	Species
Litter mass at birth	+	This study	European hare *Lepus europaeus*	[39]	Hispid cotton rat *Sigmodon hispidus*
	+			[40]	Deer mouse *Peromyscus maniculatus*
	+			[68]	European rabbit *Oryctolagus cuniculus*
Litter mass at the end of lactation	no	This study	European hare *Lepus europaeus*		
	+			[40]	Deer mouse *Peromyscus maniculatus*
Mean offspring mass at birth	no	This study	European hare *Lepus europaeus*		
	+	[42]	Reindeer *Rangifer tarandus*	[40]	Hispid cotton rat *Sigmodon hispidus*
	+	[69]	Reindeer *Rangifer tarandus*	[40]	Deer mouse *Peromyscus maniculatus*
	+	[13]	Reindeer *Rangifer tarandus*	[70]	White-footed mouse *Peromyscus leucopus*
	+	[71]	Reindeer *Rangifer tarandus*	[72]	Richardson’s ground squirrel *Spermophilus richardsonii*
	+	[73]	Red deer *Cervus elaphus*		
	+	[14]	Red deer *Cervus elaphus*		
	+	[74]	Red deer *Cervus elaphus*		
	+	[15]	White-tailed deer *Odocoileus virginianus*		
	+	[16]	Fallow deer *Dama dama*		
Mean offspring mass at the end of lactation	no	This study	European hare *Lepus europaeus*		
	+			[40]	Deer mouse *Peromyscus maniculatus*
	+			[75]	Columbian ground squirrel *Spermophilus columbianus*
Litter size at birth	no	This study	European hare *Lepus europaeus*	[68]	Norway rat *Rattus norvegicus*
	no	[76]	Guinea pig *Cavia porcellus*		
	+	[77]	Guinea pig *Cavia apere*	[78]	Meadow vole *Microtus pennsylvanicus*
	+	[79]	Flying squirrel *Glaucomys volans*	[39]	Hispid cotton rat *Sigmodon hispidus*
	+			[40]	Deer mouse *Peromyscus maniculatus*
	+			[68]	European rabbit *Oryctolagus cuniculus*
	+			[70]	White-footed mouse *Peromyscus leucopus*
	+			[80]	Richardson’s ground squirrel *Spermophilus richardsonii*
Litter size at the end of lactation	no	This study	European hare *Lepus europaeus*		
Postnatal mortality until the end of lactation	no	This study	European hare *Lepus europaeus*		
	+			[52]	Leaf-eared mouse *Phyllotis darwini*
	+			[81]	European rabbit *Oryctolagus cuniculus*

Comparison of studies investigating relationships between female body mass and reproductive parameters in precocial and altricial mammal species. Numbers refer to the studies in the reference list.

## Discussion

### Female diet and body mass effects

Our hypothesis (1), that female hares’ diet and body mass affect females’ reproductive output and success, was only partly supported by our results. Female diet (low- or high-fat) had no effect on reproductive parameters. This is in line with Hackländer et al. [45], who found no difference in litter size or mean leveret body mass at birth or at the end of lactation between the low- and high-fat diet group. However, a high-fat diet positively affects the energy budget of a female, enabling her to shorten the lactation period [45]. Hackländer et al.’s study was conducted on a subset of 18 of our 217 female hares.

Litter mass at birth was positively associated with female body mass at mating. Litter mass is a good measure of reproductive output, as it integrates litter size and intrauterine growth. Thus, our results support the assumption that females in optimal body condition show higher reproductive investment [11, 12]. In addition, our findings indicate that female European hares are, to a certain extent, capital breeders [24]. In contrast to numerous other studies of mammals, we did not find any effect of female body mass on mean offspring body mass at birth [13–16, 39, 40, 42, 69–74], or on litter size at birth [39, 40, 68, 70, 77–80].

### Seasonal effects

Our hypothesis (2), that litter mass is highest and leveret body mass is lowest in the middle of the breeding season, could only be confirmed with respect to litter mass. In contrast to the results of several mammal studies, including some on the European hare (litter size: [27–29, 46–48, 50, 82], offspring body mass: [30, 31]), we did not find any effect of season of birth on litter size or on mean offspring body mass, either at birth or at the end of lactation. In fact, our findings were very close to those of researchers working on natural populations of hares under similar climatic conditions (e.g., in the Netherlands [83]). In captive animals, a lack of effect of season on litter size and mean offspring body mass at birth can probably be attributed to the unlimited food availability over the whole year [25]. Moreover, our captive animals were partly sheltered from harsh weather conditions and were free from endoparasites. A lack of seasonal influence on litter size has also been recorded in captive Savi’s pine voles (*Microtus savii*) [84]. We suggest that the modified environmental conditions in our captive population act to reduce the strong seasonal effects that occur under continental climate conditions and mimic the conditions found in regions with an oceanic climate [85]. In line with this notion, Broekhuizen and Maaskamp [83] reported lower seasonal variation in litter size in hares in the Netherlands (mean litter size was between 1.5 and 2 in January/February, around 3 in summer and around 2.5 in autumn).

We did find a seasonal influence on litter mass at birth. This suggests that females giving birth in spring and summer invest their surplus energy in having not only more, but also heavier leverets. Litter mass at birth, which is a direct indicator of the female’s reproductive investment, seems to be unchanged by artificial conditions (including constant food availability) in captivity, and therefore may be regulated in a different way than litter size and mean offspring body mass. An explanation might be that litter mass at birth is under photoperiod control.

### Female reproductive stage and age effects

The results of the present study support our hypothesis (3), that reproductive parameters that are decisive for the reproductive performance are lower in primiparous females and in females at the end of their reproductive life, only for the parameter leveret mortality during lactation. We detected only one age-related effect on reproductive performance of European hare females in our study: leveret mortality during lactation increased with maternal age. This is in contrast to evidence of reproductive senescence causing reduced reproductive output in various mammalian species (e.g., [86–89]), including the European hare [48, 55]. Our finding that leveret mortality during lactation increases with the age of the mother suggests that maternal-effect senescence occurs in the European hare, such that senescence affects postnatal maternal care. Maternal-effect senescence has been described in another precocial mammal, the bottlenose dolphin (*Tursiops aduncus*), a species which, in contrast to the European hare, provides extensive maternal care [90]. However, our reanalysis of data published by Hackländer et al. [45] to investigate whether older females produced less daily milk than younger females showed that daily milk quantity was affected by litter size and age of leverets, but not by female age. Higher offspring mortality in primiparous females has been described repeatedly in mammals (e.g., [91–93]), and is perhaps explained by primiparous females being relatively less physically mature and less experienced in reproduction [94].

### Differences related to precociality and altriciality at birth and at the end of lactation

We did find support for a part of our hypothesis (4), that in European hares and other precocial mammals, but not in altricial mammals, female and environmental characteristics affect reproductive parameters at birth and at the end of lactation in different ways. Litter mass at birth is positively associated with female body mass in mammals, irrespective of their altricial or precocial postnatal developmental strategy (for our study see Fig 1A; for an overview of published studies see Table 3). However, we found no relationship between female body mass and litter mass at the end of lactation in our precocial hares (Fig 2B), whereas in altricial deer mice (*Peromyscus maniculatus*) female body mass is positively correlated with litter mass at the end of lactation [40]. This difference might be caused by precocial offspring feeding on solid food soon after birth and, thus, being less dependent on the mother’s body condition during lactation than altricial offspring. In hares, leverets eat solid food soon after birth, and also consume proportionally more solid food if the mother’s dietary energy content during gestation and lactation is low [95]. Thus, leverets can compensate for poor milk quality or quantity by additional foraging. However, in the natural environment this increased foraging certainly comes at the cost of increased predation risk.

Mean offspring mass at birth was not related to female body mass in our hares, but these parameters are positively correlated in several other larger precocial mammals [13–16, 42, 69, 71, 73, 74] and in all altricial mammals that are represented in our dataset ([39, 40, 70, 72]). Offspring mass at birth is negatively correlated with litter size, irrespective of postnatal development strategy (e.g., precocial: [15, 77, 96, 97], this study; altricial: [40, 52, 70, 75]). Therefore, the difference in the relationship between offspring mass at birth and female body mass is most certainly due to the difference in litter size: smaller litter sizes occur in large species (such as large ungulates) and larger litter sizes occur in small species (such as our European hare). Mean offspring mass at the end of lactation was not related to female body mass in our precocial hares, but these parameters are positively related in altricial species [40, 75]. Consistently, this might be because precocial offspring consume solid food in addition to their mother’s milk.

In line with other differences in precociality and altriciality at the end of lactation, postnatal mortality until the end of lactation was not related to female body mass in our precocial hares, whereas mortality is lower in the offspring of heavier females in altricial mammal species [52, 81].

We found no published data on maternal and environmental effects on litter size at the end of lactation in our literature search. Hence, we could not compare differences in litter size at birth and at the end of lactation in relation to precociality and altriciality. The scarcity of published data on female characteristics other than body mass and on environmental effects on other reproductive parameters at the end of lactation did not allow further comparisons between precocial and altricial mammals. We recommend that future researchers should record reproductive parameters not only at birth, but also at the end of lactation, to allow further investigation of differences between precocial and altricial mammals.

### Maternal effects on reproduction

At birth, litter size and mass in the precocial hare are strongly positively correlated with the maternal body mass, but, by the end of lactation, correlations with maternal body mass no longer exist. Litters born in spring and summer, and those born to females in the middle of their reproductive lives, are the most likely to have high litter mass and to survive lactation. In altricial mammals, reproductive parameters can be predicted from female mass, not just at birth, but also at the end of lactation. The differences of maternal effects on reproduction between altricial and precocial mammals may have a meaning for evolution. Hence, precociality might have evolved as a way of buffering offspring against maternal effects.

## Supporting information

S1 Table Overview of all models, showing response variables, predictors, random factors, scaling, numbers of females, and numbers of litters. (XLS)

S2 Table Overview of all 36 published articles in which authors provided information on reproductive parameters (i.e. litter size at birth, mean offspring mass at birth, litter mass at birth, litter size at the end of lactation, mean offspring mass at the end of lactation, litter mass at the end of lactation, offspring mortality between birth and the end of lactation) of mammals within the altricial and precocial postnatal development continuum (excluding parent-clinging mammals such as primates). (XLS)

S3 Table Overview of all 31 published articles in which authors provided information on maternal parameters (i.e. female body mass, female age, parturition sequence, lactation duration) and/or environmental characteristics (i.e. season of birth, diet availability) that could affect reproductive parameters (i.e. litter size at birth, mean young mass at birth, litter mass at birth, litter size at the end of lactation, mean offspring mass at the end of lactation, litter mass at the end of lactation, offspring mortality between birth and the end of lactation). (XLS)

S4 Table Data from this study. (CSV)
